# Development of a conditional liver tumor model by mifepristone-inducible Cre recombination to control oncogenic *kras*^*V12*^ expression in transgenic zebrafish

**DOI:** 10.1038/srep19559

**Published:** 2016-01-21

**Authors:** Anh Tuan Nguyen, Vivien Koh, Jan M. Spitsbergen, Zhiyuan Gong

**Affiliations:** 1 Department of Biological Sciences, National University of Singapore, Singapore 117543; 2 Department of Microbiology, Oregon State University, Corvallis, Oregon, USA, 97331

## Abstract

Here we report a new transgenic expression system by combination of liver-specific expression, mifepristone induction and Cre-loxP recombination to conditionally control the expression of oncogenic *kras*^*V12*^. This transgenic system allowed expression of *kras*^*V12*^ specifically in the liver by a brief exposure of mifepristone to induce permanent genomic recombination mediated by the Cre-loxP system. We found that liver tumors were generally induced from multiple foci due to incomplete Cre-loxP recombination, thus mimicking naturally occurring human tumors resulting from one or a few mutated cells and clonal proliferation to form nodules. Similar to our earlier studies by both constitutive and inducible expression of the *kras*^*V12*^ oncogene, hepatocellular carcinoma (HCC) is the main type of liver tumor induced by *kras*^*V12*^ expression. Moreover, mixed tumors with hepatocellular adenoma and hepatoblastoma (HB) were also frequently observed. Molecular analyses also indicated similar increase of phosphorylated ERK1/2 in all types of liver tumors, but nuclear localization of β–catenin, a sign of malignant transformation, was found only in HCC and HB. Taken together, our new transgenic system reported in this study allows transgenic *kras*^*V12*^ expression specifically in the zebrafish liver only by a brief exposure of mifepristone to induce permanent genomic recombination mediated by the Cre-loxP system.

Human liver cancer ranks as the fifth most prevalent malignancy and is the third leading cause of cancer mortalities worldwide with a five-year survival rate of less than 10%^1^. Liver cancer is composed of diverse and histologically distinct primary hepatic neoplasms, with hepatocellular carcinoma (HCC) as the most common type, accounting for 70-85% of approximately 0.75 million new cases yearly according to GLOBALCAN statistics^2^. The neoplastic development of human HCCs is a complex multistage process, with heterogeneity in morphology and genetics. Although major risk factors of HCC are known, a fundamental understanding of the pathophysiological and molecular mechanisms of hepatocarcinogenesis remains elusive. Several animal models for HCC, mainly in rodents, have been created and these include spontaneous models, induced models, viral models, transplantable models and lately, genetically engineered models which can closely and accurately mimic the pathophysiological and molecular features of human HCC^3^. Although rodents have offered excellent tools to cancer research, labor and cost remain a challenge in practice. The zebrafish is now emerging as an attractive model owing to the relatively low cost of maintenance, short maturation time and high number of offspring. Importantly, many of the human cancer-related phenotypes can be observed and studied in all developmental stages from the larva to juvenile to adult in zebrafish^4^. Moreover, molecular studies have suggested that the zebrafish has conserved molecular signatures during carcinogenesis when compared to those in the mammal^5^,^6^,^7^,^8^.

In our laboratory, we have used various transgenic approaches to generate several transgenic models for inducible liver tumors by inducible expression of an oncogene (*myc*, *xmrk* or *kras*^*V12*^) specifically in the liver^6^,^9^,^10^,^11^,^12^,^13^. Although these models have offered a reliable tool for many downstream studies, the inducing chemical must be continuously present throughout the course of experiments in order to maintain the transgenic oncogene expression, as withdrawal of the inducing chemical would cause repression of the transgenic oncogene and regression of the induced liver tumors. To overcome this hassle, in the present study, we made an improvement to our transgenic strategy by introducing the Cre/LoxP system to induce a permanent expression. Here, we generated two new transgenic zebrafish lines, a LexA-Cre effector line and a *kras*^*V12*^ effector line. Together with our previously generated liver-driver line to express LexPR activator^9^, we generated triple transgenic zebrafish by selective breeding to activate the oncogenic *kras*^*V12*^ gene through a brief treatment using mifepristone and demonstrated the successful generation of liver tumors of various grades. Because of frequently incomplete Cre-mediated recombination, many of the induced triple transgenic zebrafish showed distinct tumor nodules that could be due to clonal proliferation, thus resembling naturally occurring human liver tumors. Therefore, the current liver tumor model might provide a new valuable tool to analyze the development of individual liver tumors and potential chemical intervention.

## Results

### System design and generation of transgenic zebrafish for liver-specific and mifepristone-inducible Cre-recombination

**Figure 1:**
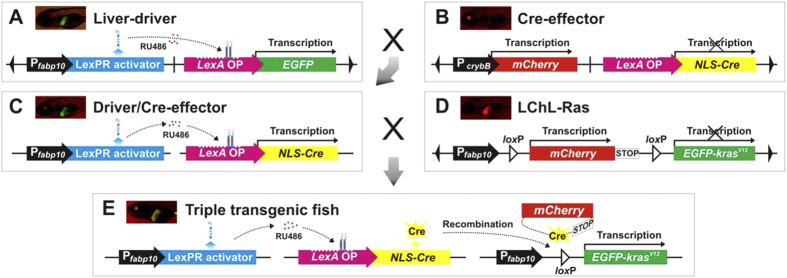
Strategies for the mifepristone-induced Cre-mediated conditional expression of *kras*^*V12*^ in transgenic zebrafish. Scheme of DNA construct and fluorescent image of transgenic fish carrying the relevant construct are shown in the same box. (**A**) Liver-driver line expressing EGFP in the liver as a transgenic reporter in the presence of mifepristone (RU486). (**B**) Cre-effector line containing a *mCherry* coding sequence as a selection marker under control of the *cryb*B promoter and a fused *NLS-Cre* gene under control of the *LexA* operator. (**C**) Double transgenic fish (Driver/Cre-effector) obtained by crossing the Liver-driver line with the Cre-effector line. (**D**) LChL-Ras line harboring a *fabp10* promoter-driven *EGFP-kras*^*V12*^ fusion gene transcriptionally interrupted by *lo*xP-flanked *mCherry* followed by a STOP cassette. (**E**) Triple transgenic fish (Triple-Tg) containing three different constructs obtained via crossbreeding of the Driver/Cre-effector line with LChL-Ras line. By applying RU486 to Triple-Tg fish, LexPR activator generated from the driver exclusively activates Cre expression in the liver, which subsequently removes DNA sequences coding *mCherry*-STOP flanked by the two *lox*P sites. After Cre-mediated recombination, the liver-specific expression of *EGFP-kras*^*V12*^ is permanently activated in the Triple-Tg fish.

A combined strategy of liver-specificity, mifepristone-inducible, and Cre/*lox*P recombination was designed to temporally control the liver-specific expression of Cre, which in turn activated the *kras*^*V12*^ oncogene to induce liver tumors in zebrafish. This approach involved the development of three transgenic lines: Liver-driver, Cre-effector and LChL-Ras with the DNA construct as illustrated in Fig. 1. The Liver-driver line has inducible liver-specific expression of the LexPR transcriptional activator by mifepristone (Fig. 1A) and has been reported previously^9^. The Cre-effector transgenic line is termed as *Tg(crybB:mCherry; LexA:Cre),* where *NLS-Cre* (nuclear localization sequence-Cre recombinase) expression was under the control of *LexA* operator. The Cre-effector also contained a selection marker, *mCherry*, which was driven by the zebrafish *cryb*B (crystallin beta B) promoter to produce red fluorescence in the lens (Fig. 1B). By crossing the Liver-driver line with the Cre-effector line, double transgenic offspring, called the Driver/Cre-effector, were obtained and capable of expressing the Cre recombinase in the liver upon administration of mifepristone (Fig. 1C). To carry out the Cre-mediated conditional expression of *kras*^*V12*^, the third transgenic line, *Tg(fabp10:loxP-mCherry-loxP-EGFP- kras*^*V12*^), abbreviated as LChL-Ras, was also generated (Fig. 1D). In this transgenic line, the *fabp10* promoter drove the loxP-flanked *mCherry* gene with a transcriptional STOP signal. The fused *EGFP- kras*^*V12*^ coding sequence was inserted downstream of the floxed *mCherry*-STOP cassette, preventing the transcription of *EGFP-kras*^*V12*^ from the upstream *fabp10* promoter. Triple transgenic fish (Triple-Tg) harboring all the three transgenic constructs, including the Liver-driver, Cre-effector and LChL-Ras, were obtained by crossing the LChL-Ras line to the Driver/Cre-effector line (Fig. 1E). Upon the administration of mifepristone, Cre was exclusively expressed in the liver of Triple-Tg fish, thus directing genomic DNA recombination. The excision of the *mCherry*-STOP cassette flanked by the two *lox*P sites resulted in an irreversible expression of *EGFP-kras*^*V12*^ in the liver. In this system, all of the constructs possess the *Ds* cis-required sequences to enhance integration of the transgene into the fish genome^14^. In this study, both Cre-effector and LChL-Ras transgenic lines were developed and the transgenic lines with single genomic insertion were selected for subsequent analyses.

### Determination of concentration- and time-dependent mifepristone induction of Cre expression

**Figure 2:**
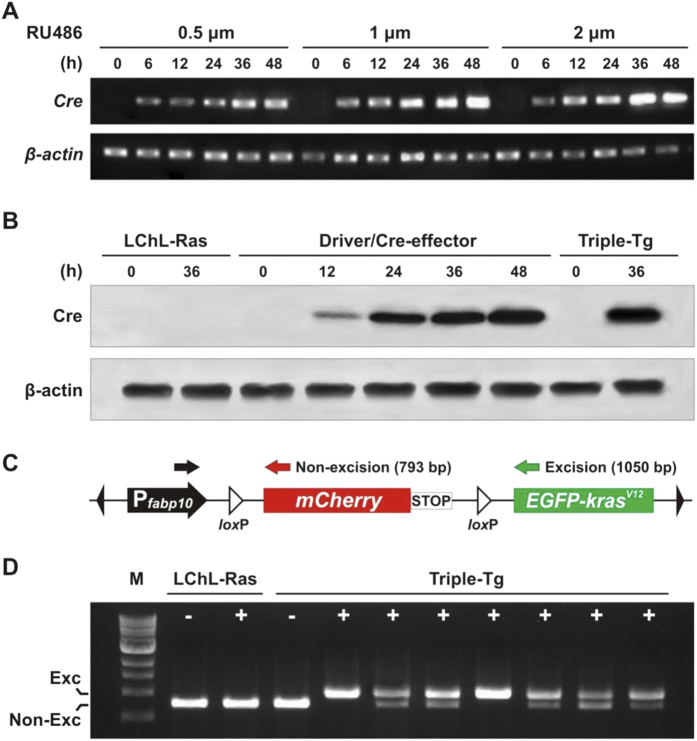
Optimization of Cre expression mediated by mifepristone in 1-month-old transgenic fish. (**A**) One-step RT-PCR determining *Cre* transcription induced by different mifepristone conditions in Driver/Cre-effector fish (n = 3 per condition). (**B**) Western blot detecting Cre protein expression in liver of 1-month-old Driver/Cre-effector and Triple-Tg fish harboring Driver, Cre-effector and LChL-Ras constructs upon 1 μM mifepristone stimulation at several durations (n = 3 fish per time-point). β-actin was used as the loading control. (**C**) Diagram of the *loxP-mCherry-loxP-EGFP-kras* construct and location of the three primers, depicted by colored arrows, used to demonstrate excision of the *lox*P-flanked sequences. A 1050-bp (base-pair) fragment amplified by two primers (black and green arrows) indicated successful Cre excision resulting in deletion of floxed *mCherry*-STOP sequences. In contrast, the combination of “black and red” primers amplifies a 793-bp fragment indicating non-excision. (**D**) PCR assay for Cre-mediated recombination using liver genomic DNA of the mifepristone-treated (+) or untreated (–) Triple-Tg and LChL-Ras fish. Non-excision showed one lower band, complete excision showed a single upper band, while incomplete excision showed both bands on the agarose gel. M stands for 1 kb DNA ladder.

To determine the expression of Cre recombinase in the Driver/Cre-effector fish, fish at one-month-old were induced for various durations with three concentrations of mifepristone (0.5 μM, 1 μM and 2 μM). Induction for 0, 12, 24, 36 or 48 hours with all three concentrations showed increasing expression of *Cre* mRNA, as determined by one-step RT-PCR (Fig. 2A). Notably, 36-48 h of induction with 1 μM and 2 μM mifepristone resulted in a high level of Cre mRNA expression. Thus, treatment with 1 μM mifepristone for 36 h was selected as an effective condition to induce Cre recombinase in 1-month-old Triple-Tg fish. To confirm the expression at the protein level under this condition, Western blot analysis was carried out and similar duration-dependent increase of Cre protein was detected from 12 h to 48 h following mifepristone induction (Fig. 2B). In contrast, Cre protein was undetectable in both untreated Driver/Cre-effector and Triple-Tg or LChL-Ras transgenics with or without induction, indicating a tight control of Cre expression in this system.

The feasibility of Cre-mediated recombination occurring at the two *lox*P sites of the *loxP-mCherry-loxP* cassette was further verified by PCR assay. At one week post-induction (wpi) with 1 μM mifepristone, liver genomic DNA was extracted from both induced and non-induced Triple-Tg fish to perform PCR with three primers as depicted in Fig. 2C. A 793-bp product was expected from non-excised transgene, while a 1,050-bp fragment was expected from excised transgene. As shown in Fig. 2D, all of the treated Triple-Tg fish (n = 22) showed efficient recombination at the DNA level, whereas no excision happened in non-treated Triple-Tg fish (n = 5). Notably, both PCR fragments were present in essentially all of the mifepristone-treated Triple-Tg fish (91%, 20 out of 22), which is likely due to the presence of non-hepatocytes in the liver and possible incomplete excision of loxP sites in the hepatocytes.

### Mosaicism of *EGFP-kras* ^*V12*^ expression in Triple-Tg fish causes hepatocellular carcinoma and other types of liver tumors

**Figure 3:**
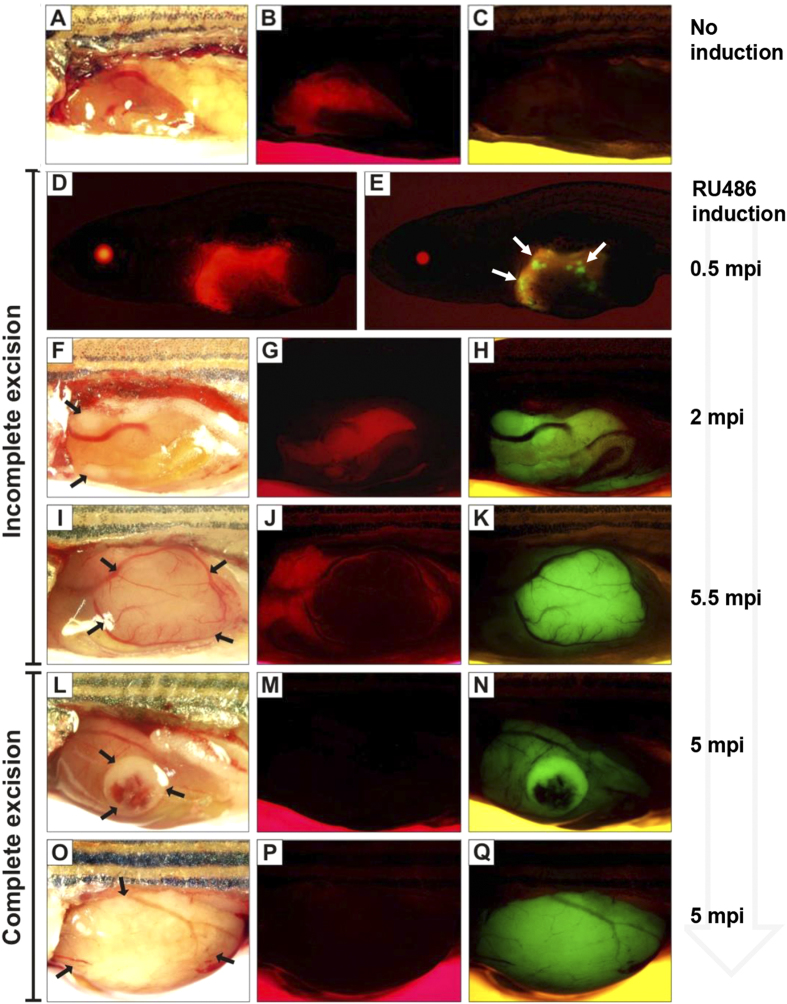
Mosaic pattern of Cre-mediated activation of EGFP-kras^V12^ in transgenic fish. Brightfield and corresponding fluorescence images of representative Triple-Tg fish are shown in the same rows. (**A–C**) Without induction, Triple-Tg fish showing normal liver morphology (**A**) with mCherry (**B**) but not *EGFP-kras*^*V12*^ (**C**). (**D–K**) Induced Triple-Tg fish at 1-month-old expressing both mCherry (**D**,**G**,**J**) and *EGFP-kras*^*V12*^ (**E**,**H**,**K**) in the liver, indicating the occurrence of incomplete Cre excision. After induction, many subsets of EGFP-positive liver cells were observed in 1.5-month-old fish (**D**,**E**). Liver tumors expressing EGFP developed in a 2.5-month-old (**F**,**G**,**H**) and 6-month-old Triple-Tg fish (**I**,**J**,**K**). (**L–Q**) Complete excision of the LChL cassettes observed in induced Triple-Tg fish at 6-month-old with the formation of large liver tumors (**L**,**O**) only expressing *EGFP-kras*^*V12*^ (**N**,**Q**) and no detectable mCherry fluorescence (**M**,**P**). Liver tumors are denoted by arrows.

As illustrated in Fig. 1, a Cre-mediated recombination event in the *loxP-mCherry-loxP* cassette can be determined by the loss of red fluorescence and the appearance of green fluorescence from the *EGFP-kras*^*V12*^ gene in the liver of mifepristone-induced Triple-Tg fish. The change in fluorescent colors was not only for marking Cre excision but also for monitoring liver tumor formation due to *kras*^*V12*^ expression. To demonstrate tumor formation in this inducible system, a cohort of 60 1-month-old heterozygous Triple-Tg fish was induced for 36 h with 1 μM mifepristone, while their non-induced Triple-Tg (n = 30) and LChL-Ras transgenics (n = 30) served as controls (Fig. 3A–C). By 2 wpi, EGFP-expressing cells appeared in multiple spots in the liver of induced Triple-Tg fish (Fig. 3D,E) and progressed to obvious liver tumors between 4–24 wpi in 65% of the induced Triple-Tg fish (39 out of 60) (Fig. 3F–K). Overall, 32/39 (82%) of these tumor fish showed partial excision as the livers exhibited both red and green fluorescence (Fig. 3D–K), whereas 7/39 (18%) of the tumor fish had complete excision (Fig. 3L–Q). Nevertheless, most of the liver tumors analyzed (93%, 40 out of 43) only exhibited EGFP expression, indicating complete excision within the tumor tissues. No EGFP-*kras*^*V12*^ expression and tumor development were observed in the controls (Fig. 3A–C).

**Figure 4:**
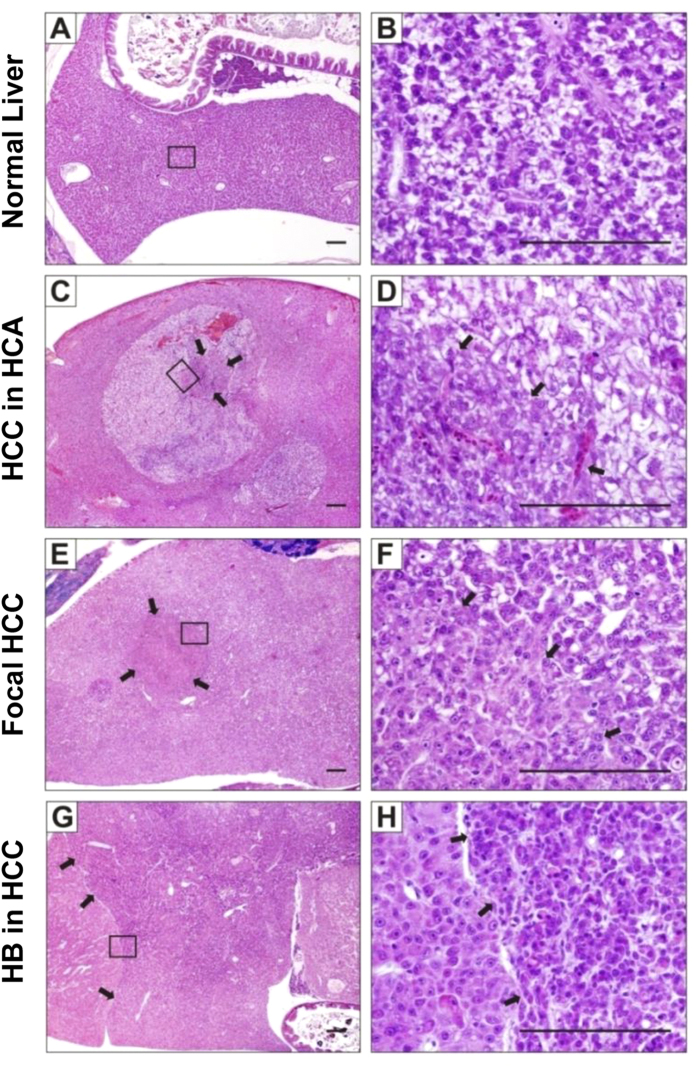
Heterogeneous liver tumors induced by *kras*^*V12*^. Histopathological examinations of liver tumors from Triple-Tg fish induced at 1-month-old. (**A**,**B**) Representative normal liver section from Triple-Tg fish without induction. (**C**,**D**) Liver tumor from induced Triple-Tg fish after 15 weeks displayed many vacuolated hepatocellular adenoma (HCA) with carcinoma grade 2 (HCC) arising in the center. (**E**,**F**) Liver tumor from induced Triple-Tg fish after 19 weeks showed HCC (grade 1) occupying 80% of the liver volume, with HCC (grade 2) arising centrally. (**G**,**H**) A Triple-Tg fish at 28 weeks after induction showing HCC (grade 2–3) involving the entire liver with extensive areas of hepatoblastoma (HB). Right panel showed high magnification of boxed area in the left panel. Arrows indicated the boundaries between different types of liver tumors. Scale bars, 100 μm.

Detailed histopathological analyses of the 39 tumor fish revealed various liver tumors, which fell into 3 main lesions including hepatocellular adenoma (HCA) (36%, 14/39), HCC (82%, 32/39) and hepatoblastoma (HB) (8%, 3/39). These observations indicated that the liver tumor spectrum was heterogeneous ranging from benign to malignant cancer with occasional HCC arising in HCA (8%, 3/39) (Fig. 4C,D), focal HCC (38%, 15/39) (Fig. 4E,F) and HB arising in HCC (8%, 3/39) (Fig. 4G,H), all suggesting ongoing liver cancer progression in induced Triple-Tg fish. Detailed histological findings from these 39 tumor fish are summarized in Supplementary Table S1. There was no histological abnormality observed in corresponding fish from the non-induced Triple-Tg and LChL-Ras transgenics control groups (data not shown).

**Figure 5:**
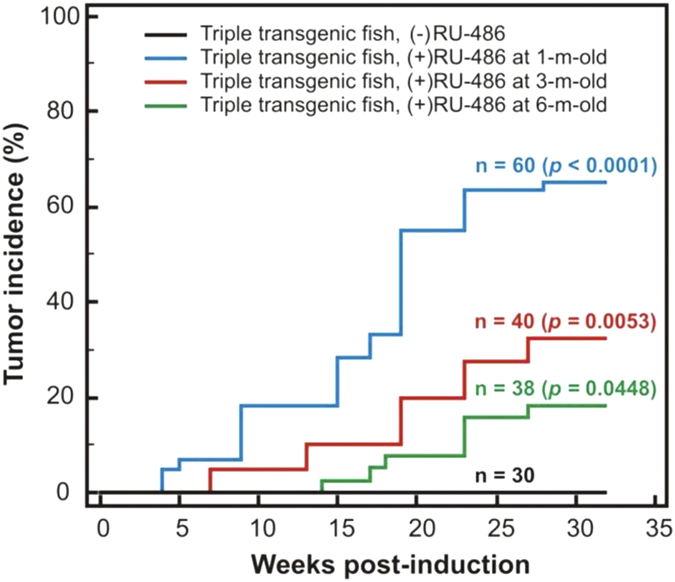
Early induction of *kras^V12^* caused high penetrance of liver tumors. Kaplan-Meier analysis identified tumor incidence and tumor induction rates of the Triple-Tg fish treated with 1 μM mifepristone (RU486) for 36 h at 1-month-old (n = 60; *p* < 0.0001), 3-month-old (n = 40; *p* = 0.0053) and 6-month-old (n = 38; p = 0.0448) as compared to untreated controls (n = 30).

To assess the influence of age on tumor incidence, two other groups of Triple-Tg fish at 3-month-old (n = 40) and 6-month-old (n = 38) were exposed to mifepristone using similar treatment conditions (1 μM mifepristone for 36 h) as the 1-month-old Triple-Tg zebrafish. Kaplan-Meier analysis revealed that the highest tumor incidence was achieved in the induced 1-month-old Triple-Tg fish (65%, 39 out of 60) as compared to induction at 3-month-old (33%, 13 out of 40) and 6-month-old (18%, 7 out of 38) (Fig. 5). Moreover, the highest rate of tumor induction was also obtained in 1-month-old treated Triple-Tg fish with a mean latency of approximately 19 weeks (*p* < 0.0001).

### Triple-Tg fish recapitulates the deregulation of ERK and Wnt/β-catenin pathways

**Figure 6:**
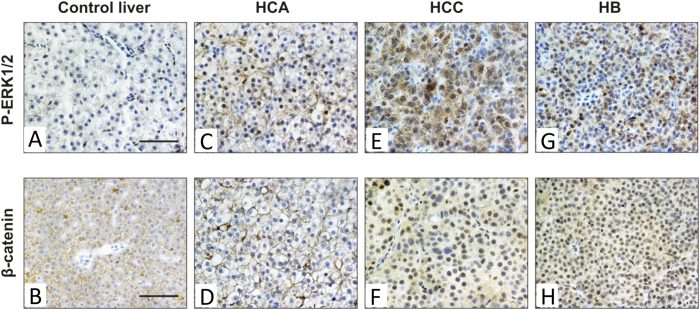
Deregulation of ERK and Wnt/β-catenin pathways in different types of *kras^V12^*-induced liver tumors. Three types of liver neoplasia including benign HCA, malignant HCC and HB were examined for the expression patterns of P-ERK1/2 and β-catenin via immunohistochemistry. (**A**,**C**,**E**,**G**) Strong mixed nuclear and cytoplasmic stainings of P-ERK1/2 were detected in three types of *kras*^*V12*^ liver tumors as compared to control liver of non-induced Triple-Tg fish. (**B**,**D**,**F**,**H**) Immunohistochemistry for β-catenin showed nuclear localization and accumulation of β-catenin only in HCC and HB, whereas normal liver and HCA displayed membranous staining of β-catenin. Scale bars, 50 μM.

In our previously reported *kras*^*V12*^ HCC models, we have found that both phosphorylated ERK1/2 and β-catenin are activated^6^,^9^. Since mosaic activation of oncogenic *kras*^*V12*^ caused various types of liver tumors in the current system, whether the molecular mechanisms of *kras*^*V12*^ -driven liver tumorigenesis might be different remains an open question. To investigate this, immunohistochemical analyses were conducted to examine the activation of the ERK and Wnt/β-catenin signaling pathways in benign and malignant *kras*^*V12*^ liver tumors. All of the liver tumor types (HCA, HCC and HB) showed ubiquitous activation of ERK1/2, as evident by intense cytoplasmic and nuclear staining of P-ERK1/2 (Fig. 6A,C,E,G). However, stronger signals of P-ERK1/2 were detected in malignant HCC and HB than HCA. Interestingly, immunohistochemistry revealed mostly membranous staining of β-catenin in HCA, which was similar to normal liver (Fig. 6B). In contrast, HCC and HB showed positive β-catenin staining only in the nuclei (Fig. 6D,F,H). These findings suggested that activation of Wnt/β-catenin, via the accumulation and translocation of β-catenin into the nuclei of hepatocytes, is required for malignant transformation in this model which is similar to our previous models in this respect^6^,^9^.

## Discussion

In this study, we have developed a new transgenic system by combining liver-specific transgenic expression, mifepristone inducibility and Cre-loxP recombination for conditional activation of an oncogene to develop inducible liver tumors in zebrafish. This approach enables the control of tumor onset to prevent developmental disorders and premature lethality which is frequently caused by constitutive expression of an oncogene^6^,^15^, thus allowing easy maintenance and propagation of the oncogene transgenic lines. Unlike our previous inducible liver tumor models where the inducing chemical is required to be present throughout the duration of experiment as withdrawal of the inducing chemical causes tumor regression^9^,^11^, the current conditional inducible system abolishes the dependence of the inducing chemical for maintenance of the tumor state, as it requires only a brief chemical induction to cause permanent genetic recombination to activate the *kras*^*V12*^ oncogene by transient induction of the Cre recombinase.

In our previous inducible liver tumor models by simple inducible expression of an oncogene in the liver, liver tumors have been uniformly induced in the whole liver with essentially 100% penetrance^9^. In contrast, in the current inducible system, only a portion of induced Triple-Tg fish form liver tumors. This is likely due to the variable levels of *kras*^*V12*^ expression in different individuals and only the fish with sufficiently high level of *kras*^*V12*^ expression are capable of developing liver cancers, as we previously reported^6^. Another notable difference in the current transgenic model is the formation of discrete tumor nodules with the co-existence of normal liver tissue. This is apparently due to the mosaic activation of oncogenic *kras*^*V12*^ because of the incomplete recombination mediated by Cre in transgenic zebrafish, as frequent co-expression of both mCherry (before excision) and Kras^v12^-EGFP (after excision) has been observed in the same liver (Fig. 3). Consistent with this, partial recombination by Cre has been reported in most previous transgenic zebrafish studies^8^,^16^,^17^,^18^,^19^. In our current transgenic system reported here, the tumor nodules are likely due to clonal amplification from single or small number of recombined cells, which is more similar to naturally occurring human tumors due to gene mutations. Thus, the ability to manipulate mosaic oncogene expression in subsets of cells within a tissue offers a unique opportunity to mimic spontaneous oncogenic mutations and clonal proliferation in humans^20^. The focal lesions varying from foci of hepatocellular alteration to hepatocellular adenoma to hepatocellular carcinoma or mixed hepatocellular carcinoma and hepatoblastoma are also quite similar when comparing the Triple-Tg zebrafish line to carcinogen-treated zebrafish. Also the evolution of individual neoplastic lesions is similar in carcinogen-treated and the Triple-Tg line in that we often saw hepatocellular carcinomas arising within hepatocellular adenomas, more anaplastic foci of hepatocellular carcinoma arising in a lower grade hepatocellular carcinoma mass, and also documented hepatoblastoma arising in mixed hepatocellular/biliary carcinomas^21^.

Previously, we have found that the level of *kras*^*V12*^ expression should be sufficiently high for generation of HCC^6^. By increasing the concentration of the chemical inducer, we can increase the level of transgenic oncogene expression and reproducibly achieve 100% penetrance in tumor production in our previous inducible transgenic models^9^. However, we contrastingly observed that only 65% of induced Triple-Tg fish induced at 1-month-old developed liver cancer, indicating that not all induced transgenic fish achieved sufficiently high level of *kras*^*V12*^ expression. By applying the same induction protocol, tumor induction rates were found to be higher in the younger group of 1-month-old Triple-Tg fish than in the older groups. This is similar to our earlier observations that younger fish are more sensitive to tumor induction than older fish, as the former requires a shorter time for onset of tumors than the latter^13^. The effect of age of the fish on the effectiveness of transgenes at inducing hepatic neoplasia is also similar to the effect of age on efficacy of carcinogens in zebrafish. We had the greatest success at inducing high incidences of liver neoplasia in carcinogen treated zebrafish when they were exposed at a young age. This finding is not surprising since cell proliferation is well recognized as a tumor promoter^21^.

Another interesting phenomenon from the new transgenic system is the production of a broad spectrum of liver tumors from HCA to HCC and HB, which may reflect different levels of *kras*^*V12*^ expression and/or additional gene mutations in different tumor nodules. Activation of Ras signaling through the ERK pathway has been firmly established as a major determinant of tumor cellular processes^22^,^23^. Recently, the development of HB has been found in Noonan syndrome patients with constitutively activated Ras/ERK signaling^24^. All these suggest the ubiquitous role of Ras signaling in the initiation and development of a wide range of hepatocellular neoplasia. The presence of HCCs in a hepatocellular adenoma and mixed liver tumors has also been observed in some human cases^25^,^26^.

So far, we have established *kras*^*V12*^-induced liver cancer models in zebrafish through three different transgenic systems, including constitutive^6^, mifepristone-inducible^9^ and mifepristone-inducible Cre/*lox*P recombination in the present study. These *kras*^*V12*^transgenic zebrafish are the first *in vivo* models to address the molecular mechanisms underlying Ras-driven liver tumorigenesis that recapitulates typical hallmarks of human HCC, thus validating our transgenic zebrafish in modeling human liver cancer. Although all of these systems efficiently induce liver tumors in transgenic zebrafish, each has its own advantages and disadvantages. In the constitutive *kras*^*V12*^ transgenic system, early Ras activation triggers heterogeneous liver tumorigenesis due to variable levels of *kras*^*V12*^ expression and frequently causes premature lethality. HCC onset in this system is relatively long (9 months) and the tumor incidence is relatively low (approximately 30%). Therefore, this transgenic line may not be practical for anti-cancer drug screening purposes but could be used instead for chemical carcinogen screens as well as tumor enhancer screens to identify mutations that accelerate the onset of Ras-induced HCC. The second mifepristone-inducible system enables us to temporally control the onset of *kras*^*V12*^-induced tumor. Because of dosage-dependent activation of *kras*^*V12*^ expression, rapid induction (within 1 month) of liver tumors with 100% penetrance is achievable. Moreover, these induced tumors can be regressed upon removal of mifepristone. Thus, the second system is ideal for investigating tumor progression and regression. In particular, this inducible system allows the development of homogenous tumor cells throughout the liver and hence provides uniform tumor materials for biochemical and molecular analyses. However, human cancer is normally initiated by a sporadic event in a single or a group of cells, where clonal expansion from these cancer cells results in tumor development. In this context, the third transgenic system via mifepristone-inducible Cre recombination could more faithfully mimic the process of sporadic tumor formation in human, as partial Cre-mediated excision results in mosaic oncogene expression in subsets of cells within a transgenic liver. A broad and heterogeneous liver tumor spectrum was obtained in this system as compared to the others. Experimentally, this system needs only a short pulse of inducer treatment and is more convenient and less laborious than the mifepristone-inducible system that requires continuous presence of inducer to maintain the expression of *kras*^*V12*^. This system could also be a better choice for screening of chemical inhibitors due to the removal of the chemical inducer (mifepristone) to reduce the potential complications from the presence of multiple chemicals. However, further optimization of the induction condition will be required to generate more uniform tumor development in the same batch of fish as the current study indicates a great variation in tumor occurrence, mortalities and tumor types.

It is well known that hepatocarcinogenesis in human is quite diversified in terms of molecular mechanisms. Many genes and molecular pathways have been reported to drive the process of hepatocarcinogenesis^27^. Other than the three oncogene-induced liver tumor models established in our laboratory^6^,^9^,^10^,^11^,^12^,^13^, several other liver tumor models have now been established in zebrafish through overexpression of other oncogenes including *edn1*, *src*, *HBx*, *HCP* (hepatitis C virus core protein), *UHRF* and β-catenin, etc^28^,^29^,^30^,^31^,^32^. Our comparative transcriptomic analyses of the *xmrk*, *Myc* and *kras* zebrafish liver tumors indicate that each of the zebrafish oncogene models represents a subset of human HCC samples, indicating the necessity of the generation of multiple oncogene tumor models for human liver cancers^33^. These zebrafish models have since emerged as powerful tools for understanding the molecular mechanisms of hepatocarcinogenesis and developing a chemical screening platform for discovery of new anti-cancer drugs^29^. For example, in the *UHRF* transgenic zebrafish model, Mudbhary *et al.* have found that overexpression of UHRF causes DNA hypomethylation and inhibits senescence, which acts as a primary means to restrict liver tumors. Similar mechanism is likely to occur in human HCC as the authors observed similar changes in clinical samples^29^.

Taken together, our new transgenic system reported in this study allows transgenic *kras*^*V12*^ expression specifically in the zebrafish liver only by a brief exposure of mifepristone to induce permanent genomic recombination mediated by the Cre-loxP system. This transgenic zebrafish may act as a useful model system to study liver cancers that arise from a single or small number of precursor cells through clonal proliferation.

## Methods

### Zebrafish maintenance

This study involving zebrafish was carried out in accordance with the recommendations in the Guide for the Care and Use of Laboratory Animals of the National Institutes of Health and the protocol was approved by the Institutional Animal Care and Use Committee (IACUC) of the National University of Singapore (Protocol Number: 096/12). Zebrafish were maintained according to established protocols, as described in our previous publications^11^,^34^. Throughout the experiments, humane endpoints were used and the experimental fish were euthanized prior to the defined experimental endpoints. The health of the fish was monitored twice a day and water quality was monitored daily. Euthanasia was conducted when fish showed signs of unrelieved sickness, pain and distress (e.g. inactive, unbalanced swimming; lack of appetite; bleeding from the gill cover and/or change of skin color, etc.).

### DNA constructs

The liver driver/reporter construct has been described previously^9^. For the Cre-effector construct, a NLS-Cre fragment was amplified by PCR from the pACN (Bunting *et al.*, 1999) using primers 5′-AGAGGAATTCCACCATGGCCAATTTACTGACCGTACACAAAATTTGC-3′ and 5′-CCTGCTGGAAGATGGCGATTAGCCATTAA-3′. The product was used as a template in the secondary PCR using primers 5′-AGAGGAATTC*CACCATGG***CACCCAAGAAGAAGAGGAAGG**TGGCCAATTTACTGACCGTACACAAAATTTGC-3′ and 5′- CCTGCTGGAAGATGGCGATTAGCCATTAAGCTTATAGCGGCCGCAGAG-3′. The product, which contained a nuclear localization sequence (bold) fused to Cre recombinase and Kozak sequence (italic), was digested with *Eco*RI/*Not*I and cloned into the same site to replace the *EGFP-kras*^V12^ portion in the Ras effector construct reported previously^9^. The LChL-Ras construct was generated by cloning the *fabp10* promoter cut at *Xho*I/*Pme*I sites into pMDS6 vector^35^ containing loxed *mCherry*-STOP cassettes with *lox*2272 sites for Cre recombinase in front of the fused *EGFP-kras*^*V12*^ sequences.

### Microinjection

Five-10 pg of plasmid DNA with 25–50 pg of *in vitro* synthesized transposase RNA was co-injected into zebrafish embryos at the one- to two-cell stage. The injected embryos were raised to adults and out-crossed to wild-type fish for testing germline transmission.

### Genotyping

Southern blot analysis was used to ensure that the transgenic fish carry a single insert. Briefly, each transgenic fish was crossed with a WT fish to obtain embryos for extracting genomic DNA, which was then digested with *Sma*I and *Xho*I and separated on a 0.7% agarose gel. Nitrocellulose membrane was used to transfer the DNA, which was then hybridized with an mCherry probe. For determination of genotypes and Cre-mediated recombination in multiple transgenic fish, genomic DNA was extracted from adult fish tail fin using the DNeasy Blood & Tissue Kit (Qiagen) according to manufacturer’s protocols. PCR was then performed using the following primers: Driver-reporter primers, 5′-AAATCATTGCCAGGTTTTCG (forward) and 5′- AGCCCTTCCAAAGGAATTGT (reverse); Cre-excision primers, 5′-GGCATTTACCTCATCTTCTGCTGG (forward) and 5′- GGTGCTCAGGTAGTGGTTGTCGG (reverse); Non-Cre-excision primers, 5′-GGCATTTACCTCATCTTCTGCTGG (forward, the same Cre-excision forward primer) and 5′- CCCATGGTCTTCTTCTGCAT (reverse).

### Western blot and immunohistochemical analyses

Immunoblotting and immunohistochemical analyses were carried out as described previously^6^,^9^. For Western blotting, samples were lysed in lysis buffer containing Complete Protease Inhibitor Cocktail (Roche) and resolved by SDS-PAGE. The separated proteins were then transferred to PVDF membrane and probed with anti-Cre (Abcam) or anti-actin (Sigma) antibodies. For immunohistochemistry, 5-μm formalin-fixed and paraffin-embedded (FFPE) tissue sections were treated prior to incubation with primary antibodies and detected using the Metal Enhanced DAB Substrate Kit (Pierce Biotechnology). Sections were then counterstained with hematoxylin, dehydrated and permanently mounted. Primary antibodies used in this study included anti-Kras (F234, Santa Cruz Biotechnology), anti-Kras-2B (C19, Santa Cruz Biotechnology), phospho-ERK1/2 (Thr202/Tyr204, Cell Signaling Technology), phospho-AKT (Ser473, Cell Signaling Technology), phospho-S6 (Ser235/236; Cell Signaling Technology) and β-actin (Sigma).

### Tumor screening and histopathological analysis

Each group of induced driver-effector double transgenic fish treated with different mifepristone concentrations was monitored under the fluorescent Nikon SMZ1600 stereomicroscope every week to determine the rate of tumor induction. All tumor-bearing fish were euthanized with 250 mg/L MS222 and dissected to open the abdominal area and photographed with a Nikon DXM1200F digital camera. Fish were then fixed in Bouin’s fixative, embedded in paraffin, sagittal sectioned, and stained with hematoxylin and eosin preceding histopathological diagnosis.

### Statistical analyses

The log-rank Kaplan-Meier analysis was used to compare rates of tumor induction between groups of induced transgenic fish. A *P* < 0.01 was chosen to be statistically significant.

## Additional Information

**How to cite this article**: Nguyen, A. *et al.* Development of a conditional liver tumor model by mifepristone-inducible Cre recombination to control oncogenic *kras^V12^* expression in transgenic zebrafish. *Sci. Rep.* **6**, 19559; doi: 10.1038/srep19559 (2016).

## Supplementary Material

Supplementary Information
