# Supplementary material for: Development of a conditional liver tumor model by mifepristone-inducible Cre recombination to control oncogenic krasV12 expression in transgenic zebrafish
Source: Sci Rep. 2016 Jan 21;6:19559. doi: 10.1038/srep19559 (PMC4726387; doi:10.1038/srep19559)
Supplement: Supplementary Information [file srep19559-s1.pdf]

Supplementary Information

Development of a conditional liver tumor model by mifepristone-inducible Cre recombination to control oncogenic *kras*^{V12} expression in transgenic zebrafish

Anh Tuan Nguyen[1, 2], Vivien Koh[1], Jan M. Spitsbergen[2], Zhiyuan Gong[1]*

[1]Department of Biological Sciences, National University of Singapore, Singapore 117543

[2]Department of Microbiology, Oregon State University, Corvallis, Oregon, USA, 97331

*Authors for correspondence (dbsgzy@nus.edu.sg)

Supplementary Table S1. Histopathologic findings in Triple-Tg zebrafish overexpressing *kras*^{V12} in liver since 1-month-old

No.	Wpi	Histological diagnoses of liver tumors
1	4	Adenoma 2 mm
2	4	Adenoma 2 mm
3	4	Adenoma 2 mm with Carcinoma arising in the center
4	5	Carcinoma involving entire liver
5	9	Adenoma 2 mm and Carcinoma 2 mm
6	9	Adenoma 2 mm
7	9	Adenoma 4 mm
8	9	Adenoma 4 mm with Carcinoma arising in the center
9	9	Adenoma 1 mm and Carcinoma 4 mm
10	9	Carcinoma involving entire liver
11	9	Carcinoma involving entire liver
12	15	Adenoma 2 mm with and Carcinoma arising in the center
13	15	Adenoma 1 mm and Carcinoma 4 mm
14	15	Carcinoma occupying 80% of liver
15	15	Carcinoma 4 mm
16	15	Carcinoma involving entire liver
17	15	Carcinoma involving entire liver
18	17	Adenoma 4 mm
19	17	Carcinoma 4 mm
20	17	Adenoma 1 mm and Carcinoma 3 mm
21	19	Carcinoma 4 mm
22	19	Carcinoma 3.5 mm
23	19	Carcinoma involving entire liver
24	19	Carcinoma involving entire liver with blood vessel invasion
25	19	Carcinoma occupying 80% of liver
26	19	Carcinoma 2 mm
27	19	Carcinoma occupying 90% of liver
28	19	Carcinoma involving entire liver with foci of Hepatoblastoma
29	19	Carcinoma involving entire liver with foci of Hepatoblastoma
30	19	Carcinoma 4 mm
31	19	Carcinoma 4 mm
32	19	Carcinoma occupying 80% of liver
33	19	Carcinoma 4 mm
34	23	Adenoma 4 mm
35	23	Adenoma 2 mm
36	23	Carcinoma 4 mm with blood vessel invasion
37	23	Carcinoma 4 mm
38	23	Carcinoma 4 mm
39	28	Carcinoma involving entire liver with foci of Hepatoblastoma
